# Optimization of Alkaline Extraction of Polysaccharides from *Ganoderma lucidum* and Their Effect on Immune Function in Mice

**DOI:** 10.3390/molecules15053694

**Published:** 2010-05-25

**Authors:** Sheng-Quan Huang, Jin-Wei Li, Zhou Wang, Hua-Xin Pan, Jiang-Xu Chen, Zheng-Xiang Ning

**Affiliations:** 1 College of Light Industry and Food Sciences, South China University of Technology, Guangzhou 510640, China; E-Mails: huangfeihong0220@sina.com (S-Q.H.); 345294613@qq.com (Z.W.); 478740826@qq.com (J-X.C.); 2 Infinitus (China) Co., Ltd., Jiangmen 529156, China; 3 State Key Laboratory of Food Science and Technology, Jiangnan University, Wuxi 214122, China; E-Mail: jwli@jiangnan.edu.cn (J-W.L.); 4 Guangzhou University of Traditional Chinese Medicine, Guangzhou 510632, China; E-Mail: gzphx@126.com (H-X.P.)

**Keywords:** *Ganoderma lucidum*, alkaline extraction, polysaccharides, yield, immune activity

## Abstract

Response surface methodology was employed to optimize the conditions for alkaline extraction of polysaccharides from *Ganoderma lucidum*. The results indicated that the optimum conditions were an extraction temperature of 60.1 °C, an extraction time of 77.3 min, a sodium hydroxide (NaOH) concentration of 5.1% and a substrate/liquid ratio of 1:21.4. Immunological assays results have shown that the alkaline soluble polysaccharides have no noticeable effects on monocyte phagocytosis and immune organ (spleen, thymus) weight of of immunocompromised mice at the tested dosages. However, they could restore delayed type hypersensitivity reaction to dinitrofluorobenzene (DFNB), hemolysis antibody levels at the three doses applied, and improve the natural killer cell activity at the high-dose and medium dose.

## 1. Introduction

Glossy ganoderma (*Ganoderma lucidum,* known as *Lingzhi* in China) is an oriental fungus and a traditional Chinese medicine that has been used for more than two thousand years to promote health and longevity The fruiting bodies, cultured mycelia and spores of *G. lucidum* are reported to be effective in the treatment of chronic hepatopathy, hypertension, hyperglycemia and neoplasia [1,2,3]. The main functional components of *G. lucidum* include polysaccharides, proteins, peptides, amino acids, triterpenes, *etc.* Recent literature showed that the polysaccharide fraction isolated from this fungus is an important functional factor that has been reported to stimulate the proliferation of mouse spleen lymphocytes [4], and to exhibit various other bioactivities, including anti-HIV, anti-herpetic, antiviral [5], immune regulating [6,7] and anti-tumor properties [8]. Bao *et al.* obtained a crude polysaccharide fraction from *G. lucidum* by hot water extraction and found that it exhibited immunostimulating activity in mice [6].

Extraction of polysaccharides is an important process for their application or further research and development, and this has prompted the publication in recent years of numerous research papers on the technology for extraction of polysaccharides from plants or fungi. Dong *et al.* optimized the hot water extraction of polysaccharides from cultured mycelium of *Cordyceps sinensis* using a Box-Behnken design [9]. Yang *et al.* employed ultrasound technology to extract polysaccharides from longan fruit pericarp and determined the optimal extraction conditions by response surface methodology. They found that the 1,1-diphenyl-2-picryl hydrazyl (DPPH) radical scavenging activity of the polysaccharides could be improved by application of ultrasound treatment [10]. Qiao *et al.* used response surface methodology to optimize the conditions for hot water extraction of polysaccharides from *Hyriopsis cumingii* [11]. Cai *et al.* studied the effects of hot water extraction parameters on the yield of polysaccharides from *Opuntia milpa alta* and established the optimal extractions conditions [12]. Wang *et al*. investigated polysaccharide extraction from *Poria cocos* with the assistance of ultrasound [13]. Wang and Ma optimized the pulsed ultrasound-assisted extraction of polysaccharides from *Ganoderma lucidum* [14]. Huang *et al.* studied the microwave-assisted extraction of polysaccharides from spores of *Ganoderma atrum* using response surface analysis [15].

In general, hot-water extraction is the most widely used technology for polysaccharide extraction, but it should be noted that hot-water extraction of polysaccharides is associated with lower yields, long extraction times and high temperatures, so it is desirable to find a novel extraction technology for polysaccharides that avoid the disadvantages of hot water extraction. Ultrasound or microwave assisted extraction can accelerate the extraction process and thus improve the extraction of bioactive compounds [16,17], and there have been numerous reports on the application of ultrasound or microwave assisted extraction in order to realize higher yields and time-savings. Nevertheless, the yield of hot-water extraction assisted by ultrasound or microwaves is only about 3%, according to the results of our preliminary experiments, and there are still plenty of polysaccharides in the water extraction residues. Kim has reported that the polysaccharides extracted by alkaline solution were composed of four kinds of monosaccharide and 18 kinds of amino acid and displayed significant anti-tumour activity [18].

The objective of the work reported in this paper was to improve the yield of polysaccharides from *Ganoderma lucidum*, using a Response Surface Methodology (RSM) design, which explores the relationships between several variables and one or more response variable and uses designed experiments to obtain an optimal response and thus optimize the alkaline extraction conditions, and further to study the immune activity of the alkaline soluble polysaccharide fraction.

## 2. Result and Discussion

### 2.1. Statistical analysis and model building

The experimental data and the process variables for yield of the crude polysaccharides under different extraction conditions are presented in Table 1.

**Table 1:** The results for response surface methodology of alkaline extraction polysaccharides from *Ganoderma lucidum.*

Number	Temperature	Time	Concentration	Alkali/ solid	Yield
	X1/ ^o^C	X2/min	X3/%	X4/mL/g	Y/%
1	50	60	4	15	5.87
2	70	60	4	15	6.25
3	50	100	4	15	6.35
4	70	100	4	15	7.01
5	50	60	6	15	5.53
6	70	60	6	15	5.23
7	50	100	6	15	6.25
8	70	100	6	15	6.46
9	60	80	5	15	7.86
10	50	80	5	20	7.63
11	70	80	5	20	7.61
12	60	60	5	20	7.76
13	60	100	5	20	7.45
14	60	80	4	20	7.74
15	60	80	6	20	7.96
16	60	80	5	20	8.12
17	60	80	5	20	8.21
18	60	80	5	20	8.04
19	60	80	5	20	8.11
20	60	80	5	20	8.14
21	60	80	5	20	8.11
22	50	60	4	25	6.48
23	70	60	4	25	6.55
24	50	100	4	25	4.95
25	70	100	4	25	5.66
26	50	60	6	25	7.21
27	70	60	6	25	6.71
28	50	100	6	25	6.18
29	70	100	6	25	6.09
30	60	80	5	25	8.09

The yield of polysaccharides varied from 4.95% to 8.21%. Bao *et al* reported that the yield of polysaccharides from *G. lucidum* using hot water extraction was 3.8% [19]. The yield by alkaline extraction is significantly higher than that extracted by hot-water. After the response surface regression (RSREG) procedure, the results of the analysis of variance, regression coefficient, along with the corresponding *p-*value, and the adequacy for the models of polysaccharides yield from the *Ganoderma lucidum* showed that the model data could adequately predict the experimental polysaccharide yield. The analysis of variance showed that this regression model was highly significant (P < 0.01) with an *F-*value of 198.23, implying a good fit between the predicted model and the experimental data. The value of 0.051 for lack of fit (P > 0.05) implied that it is not significant, compared to the pure error. The yield of polysaccharides changed significantly with all the quadratic term and linear coefficients of X_1_, X_2_ and X_4_, and five cross-terms of X_1_X_2_, X_1_X_3_, X_2_X_3_, X_2_X_4_ and X_3_X_4_. The importance of the independent variables on the yield could be ranked in the following order: extraction time (X_2_) > extraction temperature (X_1_) > the ratio of alkaline/solid (X_4_) >NaOH (sodium hydroxide) concentration (X_3_) according to the F-value of analysis of variance. By applying multiple regression analysis of Design-Expert 7.0 on the experimental data, the dependent variable and independent variable are related by the following second-order polynomial equation: Y(%)= - 43.962 + 0.832X_1_ + 0.294X_2_ + 3.621X_3_ + 0.621X_4_ + 0.001X_1_X_2_ - 0.016 X_1_X_3_ - 0.001X_1_X_4_＋0.005X_2_X_3_ - 0.005X_2_X_4_ + 0.057X_3_X_4_ - 0.006X_1_^2^ - 0.002X_2_^2^ - 0.415X_3_^2^ - 0.012X_4_^2^.

### 2.2. Influence of process variables on yield

**Figure 1:**
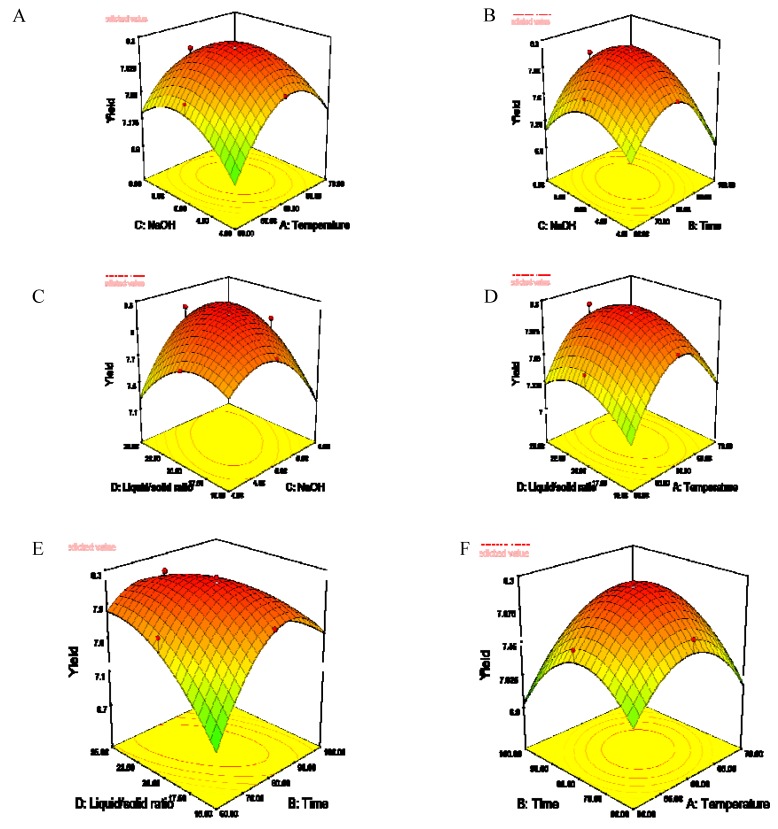
The surface and contour plot of polysaccharide yield as affected by extraction temperature (X1), extraction time (X2), NaOH concentration (X3) and ratio of liquid to solid (X4). Where (A) is X1 and X3 (liquid/solid ratio 20:1,time 80 min); (B) is X3 and X2(alkali/solid ratio 20:1,temperature 60 °C); (C) is X4 and X3 (temperature 60 °C, time 80min); (D) is X3 and X2 (NaOH 5.0%,time 80 min).,(E) is X4 and X2(temperature 60 °C, NaOH 5.0%) ;(F) is X1 and X2 (liquid/solid ratio 20:1, time 80 min).

Three-dimensional response surfaces and contour plots for the responses were plotted to study the effects of independent variables and their interactions on polysaccharide yield according to the results of the regression equations. As shown in Figure 1, the yield of polysaccharides changed significantly with the linear coefficients of extraction temperature, extraction time and ratio of alkali to solid.

In the plot of yield against temperature and NaOH concentration, the yield of polysaccharides first increased and then decreased with increasing temperature, and the highest yield was obtained at a temperature of 60 °C when the NaOH concentration was constant (Figure 1a). The yield increased with the increase of extraction time when it was below 80 min (Figure 1b). The yield increased with the increase of the ratio of alkali/solid and then decreased after the ratio was more than 20 (Figure 1c). When the extraction time and NaOH concentration were held constant, the highest yield was observed when the temperature and the ratio of alkali/solid were at the medium level (Figure 1d). Figure 1e showed that the yield increased sharply with the increase of the extraction time and then slowly decreased when the time was over 80 min and this is agreement with the results of Figure 1b. In the plot of yield against extraction temperature and time, the highest yield was observed at the temperature of 60 °C, and the yield of polysaccharides increased with increasing extraction temperature, which varied from 50 to 60 °C, and decreased with increased extraction temperature, which was varied from 60 to 70 °C. The yield of polysaccharides decreased with increasing extraction time when the temperature was less then 60 °C. The yield would decrease when the extraction temperature and time were in the higher level (Figure 1f). A medium set of extraction conditions should result in higher yield of polysaccharides. That may be due to the fact that the polysaccharides decomposed when the alkaline extraction conditions were at the high level.

### 2.3. Optimization of the alkaline extraction

By analyzing the effects of extraction conditions on the yield of polysaccharides, it was suggested that optimum extraction condition were: a temperature of 60.1 °C, a time of 77.3 min, a NaOH concentration of 5.1% and a ratio of alkaline solution to solid of 21.4mL/g, respectively. The yield of polysaccharides from *Ganoderma lucidum* was expected to be 8.21%. In order to confirm the predicted results, three further experiments using the optimum extraction parameters determined above were performed and a mean yield value of 8.30 ± 0.12% of polysaccharides was obtained, with a lower relative deviation with the predicted values.

### 2.4. Comparison in micro-structure of G. lucidum before and after alkaline extraction

**Figure 2:**
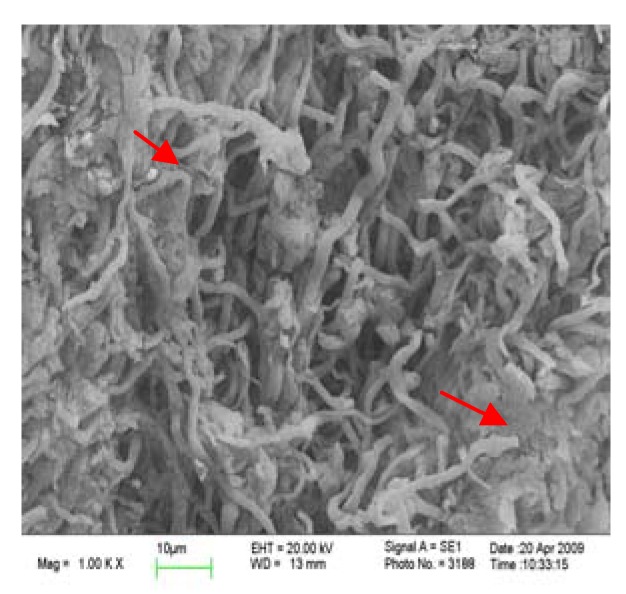
SEM micrograph of microwave/ultrasound assisted extraction residue at 1,000-fold magnification.

**Figure 3:**
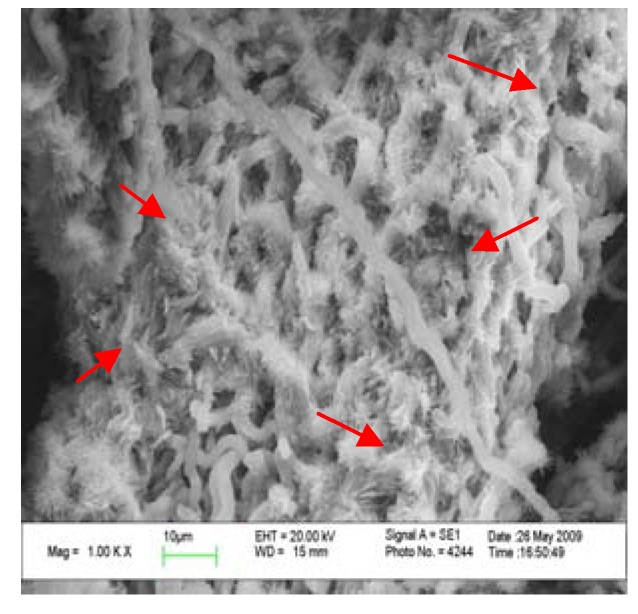
SEM micrograph of alkaline extraction residue at 1,000-fold magnification.

In order to analyze the reason for the higher yield obtained by alkaline extraction, the surfaces of gold-coated microwave/ultrasound assisted extraction residue and alkaline extraction residue from *G. lucidum* were imaged with a scanning electron microscope (Figure 2 and Figure 3).

It is easily to see from the micrographs at 1,000-fold magnification that the fibers of *G. lucidum* after the microwave/ultrasound assisted extraction treatment were broken down and the poly- saccharides could dissolve. After the alkaline extraction the coarse-fibre length was further diminished and the compact fibres were thoroughly broken down, which is good for the release of polysaccharides from the *G. lucidum* matrix.

### 2.5. Comparison in the main chemical components after the alkaline extraction

In order to confirm the scanning electron microscopy results, the main chemical components of *Ganoderma lucidum* before and after alkaline extraction were analyzed (Table 2). The contents of cellulose, hemicellulose, acid lignin and silicate after alkaline extraction were lower than without alkaline extraction. The alkaline treatment would thus result in the destruction of cell walls and breakdown of the coarse-fibre structure, which becomes looser and this is good for the release of the polysaccharides. The content of cellulose, hemi-cellulose, acid lignin and silicate from the residue decreased because of the destruction of cell wall and the fibre degradation.

**Table 2:** The main chemical components of *Ganoderma lucidum* before and after alkaline extraction.

	Dry powder/g	The extracts	The residue		
		Polysaccharides/g	Cellulose/g	Hemi-cellulose/g	Acid lignin and silicate/g
Untreated	2.000	/	0.8394	0.8526	0.0494
Microwave/ultrasound	1.6266	0.0654	0.7120	0.6934	0.0546
Microwave/ultrasound/alkaline	1.3076	0.1608	0.5856	0.6420	0.0226

Note: ultrasonic power of 550 W, temperature of 50 °C, time of 60 min and work 5 seconds, pause 5 seconds microwave power of 350 W, time of 10 min.

### 2.6. The composition analysis of alkaline soluble crude polysaccharides (ACP)

The composition of the crude polysaccharides obtained by alkaline extraction at a temperature of 60.1 °C, time of 77.3 min, NaOH concentration of 5.1% and the ratio of alkali to solid of 21.4 mL/g were analyzed. The results showed that crude polysaccharides were composed of water, proteins, reducing sugars and impurities (Table 3).

**Table 3:** Analysis of main chemical components of *Ganoderma lucidum* crude polysaccharides.

ACP	Main chemical components(%)				
	Moisture	Polysaccharide	Protein	Reducing sugar	Impurity (pigment, ash, salt)
Untreated /alkaline	20.01 ± 0.73a	54.02 ± 1.23a	2.02 ± 0.12a	2.55 ± 0.09a	21.40 ± 0.89b

Microwave/ultrasonic/alkaline	20.85 ± 0.59a	55.13 ± 0.89a	2.24 ± 0.06a	2.76 ± 0.06a	19.02 ± 1.12a

Note: Results are represented as the means 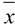 ± SD (n = 3).

The polysaccharide content was similar and ranged from 54.02% (untreated/alkali) to 55.13% (microwave/ultrasound/alkali). The protein contents ranged from 2.02% (untreated/alkali) to 2.24% (microwave/ultrasound/alkali). The main chemical components of *Ganoderma lucidum* crude polysaccharides obtained by the two treatment methods exhibited insignificant differences (p > 0.05), from each other, except for the impurities.

### 2.7. The immune activity of alkaline soluble polysaccharides in mice

The corrective clearance index, degree of ear swelling and NK cells killing activity of the model control group are significantly lower than those of the normal control group (P < 0.05), indicating that the immunocompromised model was successfully built (Table 4). The corrective clearance index of ACP was not significantly increased compared to the model control group (P > 0.05). Compared with the model control group, ACP significantly improved the degree of ear swelling (P < 0.05) for the low, medium and high-dose group, and there were no obvious differences between the ACP and pachymaran oral (P > 0.05). The results show that alkaline soluble polysaccharides are conducive to an increase of T-cell immune function in immunocompromised mice. Compared with the model control group, NK cell killing activity of ACP was significantly increased (P < 0.05) and there were no obvious differences with pachymaran oral administration (P > 0.05) for the medium-dose and high-dose groups, suggesting that ACP have a positive effect on the increase of NK cell function at the tested level. Our result is in agreement with other findings. Bao *et al*. found that the hot-water extract of *G. lucidum* spores had a stimulating effect on concanavalin A-induced mitogenic activity of T lymphocytes [6]. Cheng *et al*. investigated the effects of polysaccharide extracts of *Ganoderma capsule* on immune function in mice and found that the polysaccharide extracts of *G. capsule* possessed strong immunoregulatory effects [20].

**Table 4:** Effects of alkaline soluble polysaccharides on the carbon clearance ability, degree of ear swelling and NK cell killing activity of mice (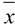 ± SD).

Groups	Dose(mg/kg/d)	The corrective clearance index-α	Degree of ear swelling(mg)	NK cells killing activity (%)
Normal control		5.28 ± 0.67^a^	14.42 ± 2.84^a^	20.78 ± 1.46^a^
Model control		4.75 ± 0.34^b^	6.75 ± 2.96^b^	16.45 ± 2.07^c^
Pachymaran oral	80	5.33 ± 0.64^a^	10.77 ± 4.04^ab^	19.74 ± 1.45^ab^
ACP	50	4.93 ± 0.36^ab^	12.22 ± 5.77^a^	17.45 ± 1.85^bc^
ACP	100	4.88 ± 0.53^ab^	14.49 ± 4.77^a^	18.75 ± 1.48^ab^
ACP	200	5.24 ± 0.58^ab^	12.12 ± 6.01^a^	19.03 ± 2.37^ab^

Note: Results are represented as the means ±SD (n = 10) for each group and there are significant differences between the values indicated with different letters in the same column. Normal control and model control were treated with equal volumes of distilled water.

The generation of serum hemolysin, and the thymus and spleen index in mice were also analyzed and the results are shown in Table 5. Compared with the normal control group, the hemolysin generation, thymus and spleen index of the model control group were significantly decreased (P < 0.05), showing that the immunocompromised model was successfully built. Serum hemolysin test could reflect the effect of polysaccharides on humoral immune response. Compared with the model control group, the hemolysin generation of each ACP-tearted group was significantly increased (P < 0.05), and there were no significant difference with pachymaran oral administration. The spleen and thymus index of all the test products showed no significant changes compared to those of the model control group.

**Table 5:** Effects of alkaline soluble polysaccharides on the generation of serum hemolysin, the thymus and spleen index in mice (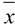 ± SD).

Groups	Dose (mg/kg/d)	OD_540nm_^*^	Spleen index	Thymus index
Normal control		1.5022 ± 0.2682^a^	48.72 ± 6.69^a^	15.90 ± 4.82^a^
Model control		0.5262 ± 0.1905^c^	38.71 ± 7.36^b^	11.05 ± 4.23^c^
Pachymaran oral	80	1.1332 ± 0.2406^b^	40.13 ± 4.19^b^	14.84 ± 4.02^ab^
ACP	50	1.3987 ± 0.2964^ab^	40.41 ± 4.00^b^	11.92 ± 4.00b^c^
ACP	100	1.1974 ± 0.4252^b^	43.64 ± 7.17^ab^	13.00 ± 2.51^abc^
ACP	200	1.2594 ± 0.2217^ab^	43.65 ± 7.98^ab^	12.48 ± 2.46^abc^

Note: Results are represented as the means ±SD (n = 10) in each group and there are significant differences indicated by the different letters in the same column. Normal control and model control were treated with equal volumes of distilled water. * The higher value of OD_540nm_ means the more generation of serum hemolysin.

Immune activity mainly includes cell-mediated, humoral and non-specific immunity. The results showed that ACP could restore delayed type hypersensitivity to DFNB at all dose levels tested. It possessed humoral immune activity by improving hemolysis antibody levels. It could improve the natural killer cell activity at the high-dose and medium-dose, which was related with the non-specific immune function. However, ACP had no noticeable effect on monocyte phagocytosis and the weight of the immune organs of immunocompromised mice in the tested dosage range. Based on its obvious immune activity, ACP has potential application as an immunomodulator additive in functional foods.

## 3. Experimental

### 3.1. Materials and equipment

*Ganoderma lucidum*, cultured from Zhejiang Longquan planting base was provided by Zhejiang Longquan Keda Agricultural Limited Company and was identified as artificial cultured *Ganoderma lucidum* by Mao Xiao-Lan of the Institute of Microbiology, Chinese Academy of Sciences. Pachymaran oral solution batch number: 20070901: 10 mL/branch, 16 mg/mL, was produced by Hunan Butian Pharmaceutical Co., Ltd. YAC-1 cells were purchased from the Cell Center of Sun Yat-sen University. All reagents were of analytical grade.

#### 3.1.1. Experimental animals

SPF-level male NIH mice (6–8 weeks old, weighing 18–22 g), were provided by the Guangdong Medical Experimental Animal Center [Permit No. SCXK (Guangdong) 2008-0002 Guangdong Monitoring and Certificate word 2008A021, Qualified Certificate No.: 0051028]. The mice were housed under controlled 12/12 hour light-dark cycle conditions and 50% relative humidity at a temperature of 25–30 °C.

#### 3.1.2. Extraction procedure

*Pretreatment*: The *G. lucidum* was ground to pass through a 2 mm screen and *G. lucidum* powder with a moisture content of 13.26% was thus obtained. The powder was refluxed twice with 95% ethanol at 70 °C in a water bath for 3 h to deactivate the endogenous enzymes and remove some soluble materials, including free sugars, amino acids and some phenols. The extracts were centrifuged (3,000 g, 10 min) according to the method of Li [21], and the *G. lucidum* pellet was vacuum-dried at 60 °C for 24 h.

*Water extraction*: The dried *G. lucidum* was suspended in water and subjected to microwave and ultrasound-assisted extraction. The ultrasound extraction was carried out for 60 min at a temperature of 50 °C, power of 550 W (sonicate for 5 seconds and pause for 5 seconds) with a JY92-2D ultrasonic generator (Xinzhi Bio-technology Institute, Shanghai, China, 20 KHz), and the microwave treatment was carried out at a power of 350 W for 10 min in a Galanz WP750 microwave oven. The remaining insoluble pellet was separated by centrifugation (5,000 g, 15 min) and at vacuum-dried 60 °C for 20 h to obtain the residue (moisture content of 12.26%) for the further alkaline extraction. The supernatant was treated according to the method of Li to obtain the water soluble crude polysaccharides [21]^.^

*Alkaline extraction*: The dried residues of *G. lucidum* were suspended in 5.1%NaOH solution to extract the polysaccharides. The extracts were centrifuged (3,000 g, 10 min) then the neutralized with 5.1%HCl. The supernatant was concentrated in a rotary evaporator under reduced pressure for about 1 h and dialyzed with running deionized water at 10 °C for 3 days. The dialysate was concentrated in a rotary evaporator at 60 °C for 30 min and washed with 100% ethanol and acetone, and finally lyophilized to obtain the alkali-soluble crude polysaccharides (ACP).

*Response Surface Methodology (RSM) Experimental Design*: RSM was used to find out the optimal alkaline extraction conditions for *G. lucidum* polysaccharides. The extraction experiments were carried out according to a central composite design with four factors and three levels. The four independent variables selected for this paper were alkaline extraction temperature, alkaline extraction time, NaOH concentration and alkaline solutionsolid ratio (Table 6). For each factor, an experimental range was based on the results of preliminary single-factor experiments. Yield of the extracted polysaccharides was the dependent variable. The complete design consisted of 30 experimental points and the experiments were carried out in a random order.

**Table 6:** Independent variables and factors levels in the response surface design.

Independent variables		Factor level		
		-1	0	+1
X1	Temperature (°C)	50	60	70
X2	Time (min)	60	80	100
X3	NaOH concentration (%)	4	5	6
X4	Liquid/solid ratio (mL/g)	15	20	25

Data from the central composite design were analyzed by multiple regression to fit the following quadratic polynomial model:

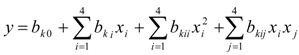
(1)
where y is the dependent variable, b_k0_, b_ki_, b_kii_ and b_kij_, are constant regression coefficients of the model, while x_i_, x_j_ are the independent variables.

#### 3.1.3. Analysis of samples

The yield of polysaccharides obtained during the extractions was calculated as the percentage of the weight of the polysaccharides to the weight of the pretreated dry powder, and the weight of polysaccharides were obtained by recalculating the sugar content results [22]. Water content was determined by weight difference after drying of samples, following the official AOAC method [23]. Sugar content was determined using the phenol–sulfuric acid colorimetric method [22]. Cellulose, hemicellulose, acid lignin and silicate were analyzed by the method of Van Soest [24]. Protein content was determined by the Coomassie Brilliant Blue G-250 method [25]. The scanning electron microscope analysis were carried out according to the following method: the sample was applied to copper stubs using double sided adhesive tape and coated with gold powders. Mounted samples were observed using a LEO1530VP field emission scanning electron microscope (LEO, Germany) at an accelerating potential of 20 kV.

### 3.2. Animal tests

The mice were randomized into six groups as follows: normal control, model control, pachymaran oral solution used as positive reference for its obvious immune activity [26], low-dose ACP with 50 mg/kg/d, medium-dose ACP with 100 mg/kg/d, and high-dose ACP with 200 mg/kg/d. The polysaccharide content of ACP (batch number is 20090608) is 55.13%. Both ACP and pachymaran were administrated by gastric feeding.

#### 3.2.1. Carbon clearance test in mice

The mice were injected intraperitoneally with dexamethasone (50 mg/kg, produced by Hubei Tianyao Pharmaceutical Co., Ltd) to cause an immunocompromised model on the 27th and 29th days of gastric feed while the normal control group was injected with an equal volume of normal saline (NS). After 31 days of gastric feed, indian ink at 0.1 mL/10 g body weight was injected into the tail vein of the mice. A total of 20 μL blood was collected through the eye orbit after 2 min (t_1_) and 20 min (t_2_), and added to 2 mL 0.1% Na_2_CO_3_ in one portion. The absorbance A (595 nm) of blood after 2 min and 20 min was measured in a normal plate reader. At the same time, mice were sacrificed by cervical dislocation, liver and spleen weights of the mice were measured. The clearance index (k) and the calibration index (α) were calculated as follows:


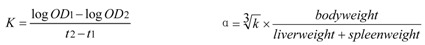


#### 3.2.2. Delayed type hypersensitivity in mice (DTH)

After the 26th day of gastric feed, mice were sensitized to 2,4-dinitrofluorobenzene solution (DNFB, Sigma) by smearing 20 μL 5% DNFB on the abdominal skin of mice with a hair removal agent. On the 27th day, mice were intraperitoneally injected with cyclophosphamide (100 mg/kg, produced by Jiangsu Hengrui Medicine Co., Ltd) to cause an immunocompromised model. On the 30th day, 20 μL of 1% DNFB was smeared on the left ear as an attack, while the right ear was smeared with 20 μL acetone solution as control. Twenty-four hours later, the DTH response to DNFB was evaluated by measuring the weight difference between the left and right ears.

#### 3.2.3. Determination of serum hemolysin test

After 27 days of gastric feed, mice were injected intraperitoneally with 5% sheep red blood cells (SRBC, 0.25 mL/10 g body weight). Serum (20 μL) was collected through the eye orbit after four more days, then, blended with 2 mL of 0.15 M NaCl saline and centrifuged for 10 min at 2,000 rpm. The supernatant (50 μL) was blended with 2.5% SRBC (250 μL) and 1:20 guinea pig alexin (250 μL). The saline was used as a control and every sample was kept in a water bath of 37 °C for 30 min, and then the reaction was terminated in ice water for 10 min. Centrifugation at 2,000 rpm for 10 min, gave 250 μL of supernatant that was measured with a microplate reader at a wavelength of 540 nm. At the same time, thymus and spleen weights of the mice were measured and the wet weights (mg) per 10 g mouse were taken as the spleen index and thymus index, respectively.

### 3.3. Determination of NK cell activity

Mice were injected intraperitoneally with dexamethasone (50 mg/kg) on the 29th and 31st day to cause an immunocompromised model. After 33 days of gastric feed, mice were sacrificed by cervical dislocation. Spleens were taken out to make spleen cell suspension conventionally. Splenocytes were adjusted to the cell concentration of 2 × 10^6^ cell/mL with RPMI-1640 medium containing 10% fetal bovine serum (FBS). YAC-1 cell were used as target for NK cell. The cells were adjusted to the concentration of 2 × 10^6^ cell/mL with PBS and stained with 1 μmol/L fluorescein dye CFSE for 10 min. The cells were adjusted to a concentration of 1 × 10^5^ cell/mL with RPMI 1640 medium.

Splenocytes prepared from the spleens of mice were used as a source of natural killer cells (NK) effector cells. The effector cells in the 96-well microplates were co-cultured with target cells (100 μL) at the ratio of 50:1. After 2 h incubation at 37 °C, cells were collected into 5 mL centrifuge tubes, mixed with 1 mL of PBS, then centrifuged at 1,200 rpm for 10 min. After discarding the supernatant and re-suspending the cells with 400 μL PBS, propidium iodide (PI, 20 μL) was added to stain them for 5 minutes, and then analyzed by flow cytometry. Taking the CFSE and PI double positive cells as the death cells, the NK cell activity was calculated according to the following formula. The natural mortality rate was the death rate of YAC-1 cells without the effector cells, while mortality rate for the test group was the death rate of YAC-1 cells with effector cells:

NK activity (%) = (test group mortality - natural mortality) / (100 - natural mortality) × 100

### 3.4. Statistics

Data are expressed in the style of mean ± standard deviation (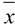 ± SD), using the statistical software SPSS 11.5 for Windows for analysis of variance, and LSD method of minimum significant differences for the comparison between groups. There are significant differences if P < 0.05.

## 4. Conclusions

We have studied the alkaline extraction of polysaccharides from *Ganoderma lucidum* and its immune activity. The five main findings can be drawn from the information presented in this paper:

1. The importance of the independent variables on the yield could be ranked in the following order: extraction time (X2) > extraction temperature (X1) > the ratio of alkaline/solid (X4) >NaOH concentration (X3).
2. The dependent variable and independent variable are related by the following second-order polynomial equation: Y(%)= -43.962 + 0.832 X1 + 0.294 X2 + 3.621 X3 + 0.621 X4 + 0.001 X1 X2 - 0.016 X1 X3 - 0.001 X1 X4 + 0.005 X2 X3 - 0.005 X2 X4 + 0.057 X3 X4 - 0.006 X12 - 0.002 X22 - 0.415 X32 - 0.012 X42
3. The optimal experimental conditions for the alkaline extraction of polysaccharides from *Ganoderma lucidum* are: a temperature of 60.1 °C, a time of 77.3 min, a NaOH concentration of 5.1% and a ratio of alkaline solution to solid of 21.4 mL/g, respectively. The yield of polysaccharides from *Ganoderma lucidum* was expected to be 8.21% and it is the 5.0 times of that of hot water extraction.
4. The scanning electron microscopy and chemical components analysis results showed that alkaline treatment could break down the fibres and accelerate the release of polysaccharides from *G. lucidum*.
5. These immunological assays results have shown that the ACP had no noticeable effect on monocyte phagocytosis and the weight of the immune organs of immunocompromised mice at the tested dosage range. However, it could restore delayed type hypersensitivity reaction to DFNB, hemolysis antibody level at all three doses applied level, and it could improve the natural killer cell activity at high-dose and medium dose.

## References

[1] Franz G. (1989). Polysaccharides in pharmacy: current applications and future concepts. Planta Med..

[2] Furusawa E., Chou S.C., Furusawa S., Hirazami A., Dang Y. (1992). Antitumor activity of *Ganoderma lucidum*, an edible mushroom, on intraperitoneally implanted Lewis Lung Carcinoma in synergenic mice. Phytother. Res..

[3] Shiao M.S., Lee K.R., Lin L.J., Wang C.T., Ho C.T., Osawa T., Huang M.T., Rosen R.T. (1994). Natural products and biological activities of the Chinese medical fungus, *Ganoderma lucidum.*. Food Phytochemicals for Cancer Prevention Ⅱ: Teas, Spices, and Herbs.

[4] Li Y.Q., Fang L., Zhang K.C. (2007). Structure and bioactivities of a galactose rich extracellular polysaccharide from submergedly cultured *Ganoderma lucidum.*. Carbohydr. Polym..

[5] Kim Y.S., Eo S.K., Oh K.W., Lee Ch.k., Han S.S. (2000). Antiherpetic activities of acidic protein bound polysaccharide isolated from *Ganoderma lucidum* alone and in combinations with interferons. J. Ethnopharmacol..

[6] Bao X.F., Zhen Y., Ruan L., Fang J.N. (2002). Purification, characterization, and modification of T lymphocyte-stimulating polysaccharide from spores of *Ganoderma lucidum*. Chem. Pharm. Bull. (Tokyo).

[7] Zhang J.S., Tang Q.J., Martin Z.K., Werner R., Fan H. (2002). Activation of B lymphocytes by GLIS, a bioactive polysaccharides from *Ganoderma lucidum*. Life Sci..

[8] Wang S.Y., Hsu H.H., Tzeng C.H., Lee S.S., Shiao M.S., Ho C.K. (1997). The antitumor effect of *Ganoderma lucidum* is mediated by cytokines released from activated macrophages and T lymphocytes. Int. J. Cancer.

[9] Dong C.H., Xie X.Q., Wang X.L., Zhan Y., Yao Y.J. Application of Box-Behnken design in optimization for polysaccharides extraction from cultured mycelium of *Cordyceps sinensis*. Food Bioprod. Process..

[10] Yang B., Zhao M., Shi J., Yang N., Jiang Y. (2008). Effect of ultrasonic treatment on the recovery and DPPH radical scavenging activity of polysaccharides from longan fruit pericarp. Food Chem..

[11] Qiao D., Hua B., Gan D., Sun Y., Ye H., Zeng X. (2009). Extraction optimized by using response surface methodology, purification and preliminary characterization of polysaccharides from *Hyriopsis cumingii*. Carbohydr. Polym..

[12] Cai W., Gu X., Tang J. (2008). Extraction, purification, and characterization of the polysaccharides from Opuntia milpa alta. Carbohydr. Polym..

[13] Wang Y., Cheng Z., Mao J., Fan M., Wu X. Optimization of ultrasonic-assisted extraction process of *Poria cocos* polysaccharides by response surface methodology. Carbohydr. Polym..

[14] Wang X., Ma H.L. (2007). Pulsed Ultrasonic-assisted extraction of polysaccharides from *Ganoderma lucidum*. Chin. Food Sci. Technol..

[15] Huang P., Xie M., Nie S., Chen Y., Li C., Xie J. (2007). Study on Microwave-assisted Extraction of Polysaccharides from Spores of *Ganoderma atrum* with Response Surface Analysis. Chin. Food Sci..

[16] Bonrath W. (2004). Chemical reactions under ‘non-classical conditions’, microwaves and ultrasound in the synthesis of vitamins. Ultrason. Sonochem..

[17] Zhang L., Liu Z. (2008). Optimization and comparison of ultrasound/microwave assisted extraction (UMAE) and ultrasonic assisted extraction (UAE) of lycopene from tomatoes. Ultrason. Sonochem..

[18] Kim B.K., Chung H.S., Chung K.S., Yang M.S. (1980). Studies on atineoplastic components of Korean basidiomycetes. Korean J. Mycol..

[19] Bao X.F., Wang X.S., Dong Q., Fang J.N., Li X.Y. (2002). Structural features of immunologically active polysaccharides from *Ganoderma lucidum*. Phytochemistry.

[20] Cheng J.W., Wu X.Q., Sun P.H., Wu Q.Q., He L., Fu L.Z., Hu C.J., Li H.B., Wei H.L. (2009). Effects of the Polysaccharide Extracts of Ganoderma Capsule on Immune Function in Mice. Edible Fungi China.

[21] Li J., Ding S., Ding X. (2007). Optimization of the ultrasonically assisted extraction of polysaccharides from *Zizyphus Jujuba cv*. Jinsixiaozao. J. Food Eng..

[22] Dubois M., Gilles K.A., Hamilton J.K., Rebers P.A., Smith F. (1956). Colorimetric method for determination of sugars and related substances. Anal. Chem..

[23] Association of Official Analytical Chemists (1995). Official Methods of Analysis.

[24] Van S.P.J., Robertson J.B., Lewis B.A. (1991). Methods for dietary fiber, neutral detergent Fiber, and Nonstarch Polysaccharides in Relation to Animal Nutrition. J. Dairy Sci..

[25] Bradford M. (1976). A Rapid and Sensitive Method for the Quantitation of Microgram Quantities of Protein Utilizing the Principle of Protein-Dye Binding. Anal. Biochem..

[26] Xie G., Wang F., Yang Z., Wang S., Zhang F., Chen Z., Fang F. (2009). Enhanced immune effects of pachymaran on inactivated influenza virus vaccune. Life Sci. Res. (China).

